# Use of antidepressants and anxiolytics in early pregnancy and the risk of preeclampsia and gestational hypertension: a prospective study

**DOI:** 10.1186/s12884-019-2285-8

**Published:** 2019-04-30

**Authors:** Nathalie Bernard, Jean-Claude Forest, George M. Tarabulsy, Emmanuel Bujold, Damien Bouvier, Yves Giguère

**Affiliations:** 1 Centre de recherche du CHU de Québec-Université Laval, 10 rue de L’Espinay, Québec City, Québec G1L 3L5 Canada; 2 Department of Molecular Biology, Medical Biochemistry and Pathology, Faculty of Medicine, Université Laval, Québec City, Canada; 3 School of Psychology, Université Laval, Québec City, Canada; 4 Department of Reproduction, Obstetrics and Gynecology, Faculty of Medicine, Université Laval, Québec City, Canada; 5 Department of Biochemistry and Molecular Genetics, CHU Clermont-Ferrand, and UCA, CNRS, INSERM, GReD, Clermont-Ferrand, France

**Keywords:** Risk of preeclampsia, Antidepressants, Anxiolytics, Pregnancy, women’s health

## Abstract

**Background:**

We investigated the association between antidepressant and anxiolytic exposure during the first and early second trimester of pregnancy (< 16 weeks), and hypertensive disorders of pregnancy (including preeclampsia and gestational hypertension) in women with singleton pregnancy.

**Methods:**

This study is based on a large prospective cohort of 7866 pregnant women. We included pregnant women aged 18 years or older without chronic hepatic or renal disease at the time of recruitment. Participants lost to the follow-up, with multiple pregnancies and pregnancy terminations, miscarriages or fetal deaths before 20 weeks of gestation were excluded from the study, as well as women with no data on the antidepressant/anxiolytic medication use during pregnancy. Information concerning antidepressant or anxiolytic medication use was extracted from hospital records after delivery. The associations between their use and the risk of gestational hypertension or preeclampsia were calculated.

**Results:**

The final sample for analysis included 6761 participants including 218 (3.2%) women who were exposed to antidepressant and/or anxiolytic medication before the 16th week of gestation. Forty-one women had a non-medicated depression or anxiety during the pregnancy. Moreover, 195 (2.9%) and 122 (1.8%) women developed gestational hypertension and preeclampsia respectively. When compared to women unexposed to antidepressant/anxiolytic medication, depression and anxiety, those using antidepressant and/or anxiolytic drugs before the 16th week of gestation were at increased risk of preeclampsia (adjusted odd ratio (aOR) 3.09 [CI_95%_ 1.56–6.12]), especially if they continued their medication after the 16th week (aOR 3.41 [CI_95%_ 1.66–7.02]) compared to those who did not (1.60 [CI_95%_ 0.21–12.34]).

**Conclusions:**

Women exposed to antidepressant and/or anxiolytic medication before the 16th week of pregnancy have a 3-fold increased risk for preeclampsia when compared to women unexposed to antidepressant/anxiolytic medication, depression and anxiety. Also, our results suggested that women who stopped their medication before the 16th week of pregnancy could be benefit from reduced preeclampsia risk.

## Background

Hypertensive disorders of pregnancy (HDP) are leading causes of maternal and perinatal morbidity and mortality. Gestational hypertension (GH) is defined as hypertension that develops for the first time at ≥20 weeks (wks) of gestation, while preeclampsia (PE) is defined as GH with one or more of the following: new proteinuria or adverse conditions (such as elevated serum creatinine, visual symptoms, oligohydramnios,…) or severe complications (such as eclampsia, hepatic dysfunction, stillbirth,…) [1]. PE affects 2–8% of pregnancies worldwide [2], but its prevalence varies between populations and is influenced by factors such as maternal and socioeconomic characteristics and ethnicity [3, 4]. Although clinical manifestations occur after 20 weeks of pregnancy, it is now well recognized that PE pathophysiological modifications at the placental level begin during the first trimester.

The prevalence of perinatal depression and anxiety disorders is estimated to vary widely from 5% to more than 25% of pregnant women and new mothers [5–9]. Depression is one of the most common complications of the prenatal and postpartum periods [7, 10]. During pregnancy, antidepressants are among the most frequently prescribed medications [11, 12]. The frequency of antidepressant use in pregnant women varies worldwide, ranging from 1.8% to up 10% [13–17]. During the pregnancy, the prevalence of benzodiazepine use, an anxiolytic, is around 5% [12, 18, 19].

Maternal mood and anxiety disorders are associated with an increased risk of poor obstetrical outcomes, including both GH and PE [20–23]. Moreover, it has also been observed that antidepressant exposure during pregnancy may increase susceptibility to hypertensive disorders during pregnancy [24, 25]. Some results suggest that women who use antidepressants in pregnancy are at increased risk for GH and PE (adjusted odds ratio between 1.30–4.86) [26–30], while other studies do not find such an association [31]. The timing of antidepressant medication exposure may be an important factor of HDP risk [26, 29]. For example, one study reported that antidepressant use between 13 and 20 weeks of pregnancy increases PE risk [26]. A recent systematic review reported that although some studies have suggested a moderately increased risk of HDP associated with antidepressant exposure, the current data do not allow a definitive conclusion on this topic [32]. Relatively few studies are available and these studies have many methodological limitations [32]. The literature on benzodiazepine use in pregnancy is dominated by studies on the risk of fetal malformations. The association between HDP risk and anxiolytic medication exposure has been explored by few studies [18, 30]. In a recent study, benzodiazepine use during pregnancy increased the HDP risk, but the association was not significant in adjusted models [30].

In this exploratory study, we use a large pregnancy cohort to investigate the association between antidepressant and anxiolytic exposure, separately and in combination, during the first and early second trimester (< 16 weeks of gestation), and HDP, including PE and GH, in women with a singleton pregnancy.

## Methods

### Study design

This study is based on a large prospective cohort on pregnancy complications that includes 7866 pregnant women recruited at the CHU de Québec-Université Laval from April 2005 to March 2010 during their first prenatal visit (median 15 wks (interquartile range 14°–15^4^ wks) to an institutional perinatal clinic. Pregnant women aged 18 years or older without chronic hepatic or renal disease were eligible to participate in the study. Exclusion criteria for the present study included women lost to follow-up, multiple pregnancies and pregnancy terminations (voluntary or medical interruption of pregnancy (VIP or MIP)), miscarriages or fetal deaths before 20 weeks of gestation. Women with no data on the antidepressant/anxiolytic medication use during pregnancy were also excluded. Women who were taking other medication for psychiatric problems such as anticonvulsant, antiepileptic, antipsychotic, stimulant or unidentified medication were excluded from the study as well as women who used antidepressant/anxiolytic in combination with antipsychotics. Details of the original study design can be found elsewhere [33–35]. Participants gave written informed consent and the study was approved by the CHU de Québec-Université Laval Ethics Review Board (initial approval date: 9 November 2004, Project 5–04–10-01 [95.05.17 l SC12–01-159).

Documentation on antidepressant (selective serotonin reuptake inhibitors (SSRI), serotonin norepinephrine reuptake inhibitors (SNRI), tricyclic antidepressants (TCA), or other antidepressants) or anxiolytic (benzodiazepine or other anxiolytic) medication was obtained following delivery from a standardized prenatal follow-up form (gynecological and obstetrical history, presence of diseases or disorders, medications,…) filled at each prenatal visit by the nurse and the physician and included in the hospital records. The period of exposure during pregnancy was recorded (< 16 wks; between 16 and 28 wks; > 28 wks). The exposure was recorded until the last documented clinical pregnancy follow-up visit. Exposure to all antidepressant or anxiolytic medication was considered. The users were subdivided in two groups: women who stopped their medication before the 16th week and those who continued their medication after the 16th week.

Diagnosis of hypertensive disorders of pregnancy was made by a senior obstetrician according to the Society of Obstetricians and Gynaecologists of Canada classification based on information retrieved from medical records. Gestational hypertension was defined as de novo hypertension (systolic blood pressure ≥ 140 mmHg and/or diastolic blood pressure ≥ 90 mmHg) after 20 weeks of pregnancy. Preeclampsia was defined as GH with proteinuria (≥300 mg in a 24-h urine collection or ≥ 2+ on dipstick in a random sample) or pre-existing hypertension and new or worsening proteinuria.

### Statistical analyses

Analyses were performed for each pregnancy outcome, PE or GH, separately. Characteristics of mothers who developed an adverse pregnancy outcome of interest and those who did not were compared using the z-test for categorical variables and the Mann-Whitney U-test for continuous variables because the assumption of normality was not met. Continuous variables are expressed as mean ± one standard deviation (SD).

Factors that were considered as potential confounders for PE and GH were based on the current literature and biological plausibility, and included: maternal age, parity (para 0, para> 0), pre-pregnancy body mass index (BMI), presence of pre-pregnancy high blood pressure (HBP) (Yes, No, Unknown), ethnicity (2 Caucasian parents, No 2 Caucasian parents, Unknown), mean arterial pressure (MAP) at the first trimester visit, maternal smoking during pregnancy (Smoker, Ex-smoker, Non-smoker, Unknown), a past history of HDP (Yes, No, Unknown), and the presence of gestational diabetes (GDM). The covariables were included in the model, whether they were significantly different or not. The pattern on missingness was assumed to be missing at random (MAR). For continuous variables, missing values were estimated by multiple imputation algorithm, (using Markov Chain Monte Carlo, 5 imputations). There were no missing data in the following potential explicative variables: maternal age at delivery, parity, presence of GDM, antidepressant/anxiolytic exposure. Less than 10% of the data were missing for the other covariates, except for pre-pregnancy BMI (overall 13.3%, in PE cases: 4.9%; in GH cases: 1.5%).

Logistic regression analyses adjusting for potential confounding factors were used to estimate the risk of GH or PE associated with exposure to anti-depressants and/or anxiolytics before 16 weeks of gestation (adjusted odds ratios [aOR] with 95% confidence intervals [CI]). With an α = 0.05, the power of the study was 80.4% for PE analysis. A *P* value of less than 0.05 was considered significant. Statistical analyses were performed using XLSTAT (2018.5 version, Addinsoft).

## Results

**Fig. 1:**
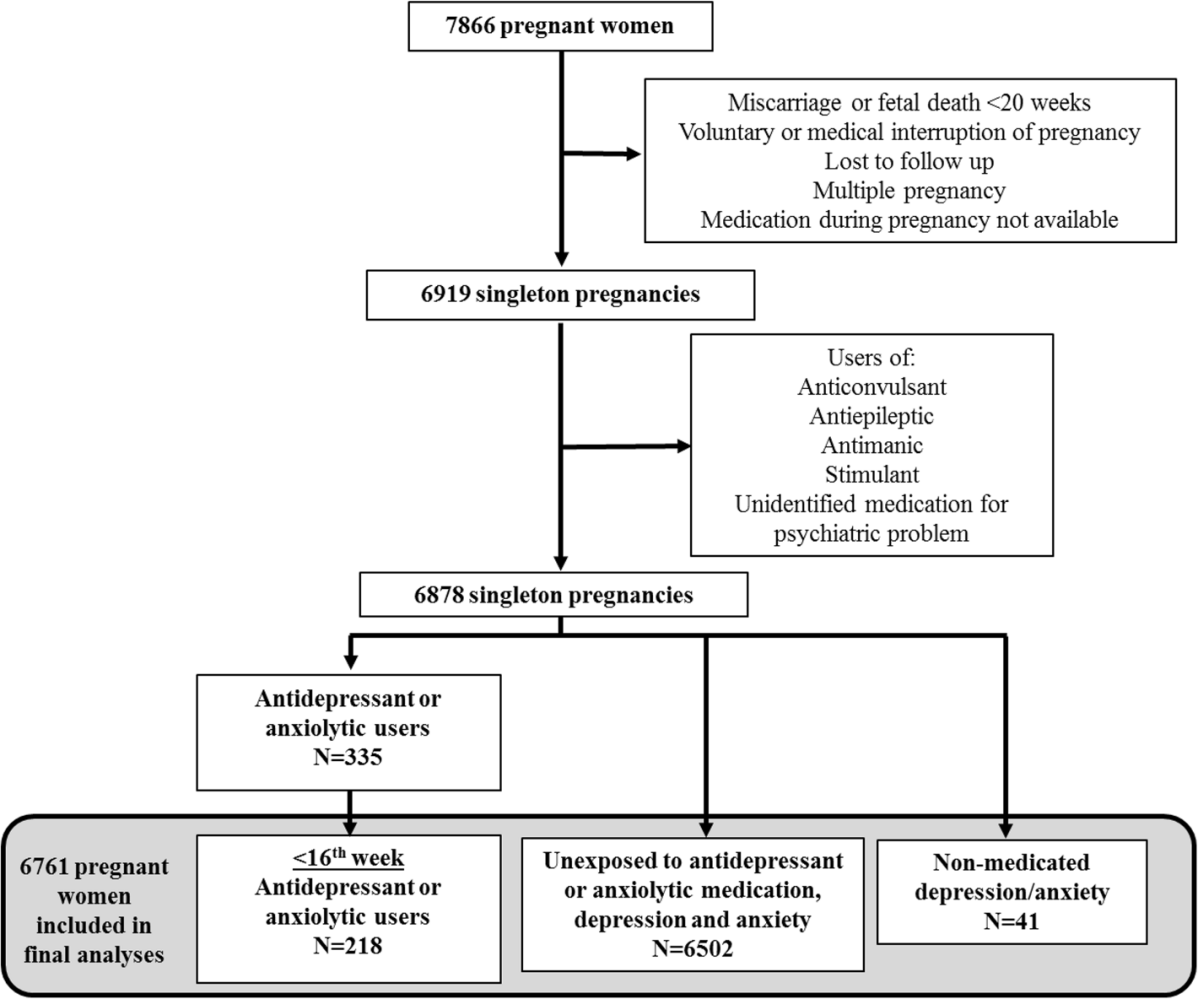
Flowchart of the study

The number of women who agreed to participate in our large prospective study was very high, with a recruitment rate of 86%. Of the 7866 participants of the prospective study, 6878 pregnant women met our inclusion criteria, of whom 335 (4.9%) were exposed to antidepressants and/or anxiolytic drugs at some point during pregnancy and 218 of them were exposed before the 16th week of pregnancy (Fig. 1). Among these 218 women, 167 continued using antidepressant and/or anxiolytic medication for at least another trimester (149/167 were still users in the third trimester). Forty-one women had a non-medicated depression or anxiety during the pregnancy. Among the 6878 pregnant women, 202 (2.94%) and 127 (1.85%) women developed GH and PE respectively. These rates are similar to those observed in the Quebec City area in another independent study [36]. Since PE pathophysiological modifications begin during the first trimester, we studied these 218 antidepressant/anxiolytic users who began medication before the 16th week and compared them to women unexposed to antidepressant/anxiolytic medication, depression and anxiety for the detailed analysis (Fig. 1). By limiting the present study to exposure before the 16th week, we wanted to ensure that antidepressant/anxiolytic exposure during the pregnancy began before the HDP diagnosis. A total of 6761 pregnancies (6474 women) were studied. Of note, none of the women contributing more than once in the cohort are found in the subgroup exposed to antidepressant/anxiolytic and who developed HDP.

Participant characteristics are presented in Table 1. Maternal age at delivery and ethnicity were not significantly different between subgroups. In comparison to women without HDP, those who developed GH or PE had significantly higher pre-pregnancy BMI and higher MAP at first visit. Furthermore, nulliparity, pre-pregnancy hypertension, GDM and a past history of HDP were significantly more frequent in women who developed HDP while smoking was less frequent.

**Table 1:** Characteristics of the study participants

	Women without HDP *n* = 6444	PE women *n* = 122	GH women *n* = 195
Maternal age at delivery^a^ (years)	29.96 ± 4.32	30.00 ± 4.68	29.90 ± 4.27
% Para 0	46.21%	68.85%†	67.69%†
% Smokers during pregnancy	13.09%	7.9%	8.15%‡
% 2 Caucasian parents	96.95%	93.91%	98.32%
Pre-pregnancy BMI (kg/m^2^)^a^	24.10 ± 5.13	27.61 ± 6.75†	27.34 ± 6.38†
% Pre-pregnancy hypertension	1.10%	11.67%†	9.28%†
MAP at the first visit (mmHg)	83.07 ± 7.86	89.10 ± 9.21†	91.27 ± 9.42†
Gestational age at delivery^a^ (wk)	39.44 ± 1.52	37.79 ± 2.09†	39.15 ± 1.38†
% GDM	6.78%	14.75%‡	13.84%‡
% Past history of HDP	3.80%	14.88%‡	18.84%†
% antidepressant and/or anxiolytic users during the first trimester	3.07% (*n* = 198)	9.02%‡ (*n* = 11)	4.62% (*n* = 9)
% Non-medicated depression or anxiety	0.59% (*n* = 38)	1.64% (*n* = 2)	0.51% (n = 1)

^a^mean ± SD

compared to women without HDP: †*p* < 0,001; ‡p < 0,05

*BMI* body mass index, *GDM* gestational diabetes mellitus, *GH* gestational hypertension, *HDP* hypertensive disorders of pregnancy, *MAP* mean arterial pressure, *PE* preeclampsia

### Use of antidepressants and anxiolytics before the 16th week of pregnancy

Women using antidepressants and/or anxiolytics during the first and early second trimester were characterized by older age and higher BMI than those who did not, they were more likely to be smokers, to be multiparous and to present a history of pre-pregnancy hypertension. Table 2 shows exposure to each class of drugs: SSRI were the most prevalent (48.5%), followed by SNRI (27.0%) and benzodiazepine (17.8%).

**Table 2:** Prevalence of classes of medication before the 16th week of pregnancy

Class of medication	Number of users (*n* = 218)^a^
Selective serotonin reuptake inhibitors (SSRI)	117 (48.5%)
Serotonin norepinephrine reuptake inhibitors (SNRI)	65 (27.0%)
Tricyclic antidepressant (TCA)	8 (3.3%)
Others antidepressants	8 (3.3%)
Benzodiazepine	43 (17.8%)

^a^19 women used 2 different classes and 2 women used 3 different classes

The proportion of antidepressant and/or anxiolytic users before the 16th week of pregnancy was significantly greater in the PE subgroup (9.0% vs 3.1% in women without HDP; *p* = 0.03). Among the 11 preeclamptic women using antidepressants or anxiolytics, 10 were antidepressant users, and one used anxiolytics. Also, among these 11 users, 10 had continued their medication until the third trimester (including one who had changed antidepressant class and added an anxiolytic medication). Analysis in more frequent types of drug exposure (SSRI, SNRI, TCA, benzodiazepine, SSRI and benzodiazepine simultaneously) were performed. Women exposed to SSRI or SNRI had a significantly higher risk of preeclampsia. Since in each class PE risk tended to increased (Table 3) and that PE frequency was low, we combined all antidepressant and anxiolytic drug exposure classes as one comparison group.

**Table 3:** Risk of HDP in antidepressant and/or anxiolytic users before the 16th week of gestation according to class of medication

	Adjusted OR (CI 95%)^a^	
	PE (*n* = 122)	GH (*n* = 195)
Unexposed (*n* = 6502)	1	1
Class of medication		
Selective serotonin reuptake inhibitors (SSRI) (*n* = 103)	3.09 (1.22–7.85) *p* = 0.018	0.75 (0.19–2.91) *p* = 0.68
Serotonin norepinephrine reuptake inhibitors (SNRI) (*n* = 57)	6.46 (2.49–16.78) *p* < 0.0001	2.83 (0.98–8.11) *p* = 0.054
Benzodiazepine (benzo)(*n* = 26)	2.64 (0.41–16.86) *p* = 0.31	0.44 (0.02–10.78) *p* = 0.41
SSRI and benzo simultaneously (n = 10)	1.44 (0.03–64.19) *p* = 0.85	2.35 (0.21–26.69) *p* = 0.49
Tricyclic antidepressant (TCA) (n = 5)	7.36 (0.22–251.11) *p* = 0.27	4.50 (0.13–159.47) *p* = 0.41
Any Antidepressant and/or anxiolytic (*n* = 218)	3.09 (1.56–6.12) *p* = 0.001	1.39 (0.66–2.92) *p* = 0.38
Non medicated depression/anxiety during pregnancy (*n* = 41)	2.92 (0.67–12.73) *p* = 0.15	0.77 (0.10–5.78) *p* = 0.80

^a^adjusted for pre-pregnancy BMI, pre-pregnancy hypertension, maternal age, ethnicity, parity, smoking during pregnancy, MAP at the first visit, past history of HDP, presence of GDM

*HDP* hypertensive disorders of pregnancy, *CI* confidence interval, *GH* gestational hypertension, *PE* preeclampsia

Women who started using antidepressant and/or anxiolytic drugs before 16th week of their pregnancy had a significant increased risk to develop preeclampsia (odds ratio (OR) 3.16 [CI_95%_ 1.68–5.98]; *p* = 0.0004), which remained similar after adjustment for the potential confounders (aOR 3.09 [CI_95%_ 1.56–6.12]; *p* = 0.001) (Table 4). Women who continued use of antidepressant/anxiolytic medication after the 16th week of their pregnancy (*n* = 167) had a greater risk of PE (OR 3.41 [CI_95%_ 1.67–7.02]; p = 0.001) compared to those who were not exposed to the antidepressant/anxiolytic medication, depression or anxiety. The PE risk of women who stopped their medication before 16 weeks of gestation (*n* = 51) was not significantly different (aOR 1.60 [CI_95%_ 0.21–12.34]) to those who were not exposed to the antidepressant/anxiolytic medication, depression and anxiety.

**Table 4:** Risk of HDP in antidepressant and/or anxiolytic users before the 16th week of gestation

Antidepressant and/or anxiolytic exposure	OR (CI 95%)	Adjusted OR (CI 95%)*
No (*n* = 6502)	1	1 PE (*n* = 111); GH (*n* = 186)
Before the 16th week of pregnancy (*n* = 218)	PE risk 3.16 (1.68–5.98)†	PE risk (*n* = 11) 3.09 (1.56–6.12)‡
	GH risk 1.53 (0.77–3.02)	GH risk (*n* = 9) 1.39 (0.66–2.92)
ᅟContinued their medication after the 16th week ᅟ(*n* = 167)	PE risk 3.80 (1.95–7.40)†	PE risk (*n* = 10) 3.41 (1.66–7.02)†
	GH risk 1.57 (0.72–3.39)	GH risk (*n* = 7) 1.28 (0.56–2.96)
ᅟStopped their medication before the 16th week ᅟ(*n* = 51)	PE risk 1.19 (0.16–8.67)	PE risk (*n* = 1) 1.60 (0.21–12.34)
	GH risk 1.40 (0.34–5.80)	GH risk (*n* = 2) 1.93 (0.42–8.92)
Non medicated depression/anxiety during pregnancy (*n* = 41)	PE risk 3.00 (0.71–12.58)	PE risk (*n* = 2) 2.92 (0.67–12.73)
	GH risk 0.88 (0.12–6.47)	GH risk (*n* = 1) 0.77 (0.10–5.78)

*adjusted for pre-pregnancy BMI, pre-pregnancy hypertension, maternal age, ethnicity, parity, smoking during pregnancy, MAP at the first visit, presence of GDM

*HDP* hypertensive disorders of pregnancy, *CI* confidence interval, *GH* gestational hypertension, *PE* preeclampsia

†*p* < 0.001; ‡*p* = 0.01

In unadjusted and adjusted analysis, the GH risk was increased but not reached statistical significance. The risk of GH was not significantly changed if the exposure to the medication continued after or stopped before the 16th weeks (Table 4). The women with a non-medicated depression or anxiety were at increased PE risk, but the association did not reach significance. Detailed analyses of the characteristics of PE women are presented in Table 5.

**Table 5:** Characteristics of the preeclamptic women

	Antidepressant and/or anxiolytic users	
	No	Yes
	(*n* = 111)	(*n* = 11)
Maternal age at delivery^a^ (years)	29.76 ± 4.43	32.46 ± 6.46
% Para 0	69.37%	63.64%
% Smokers during pregnancy	6.73%	20,00%
% 2 Caucasian parents	93.27%	100,00%
Pre-pregnancy BMI (kg/m^2^)^a^	27.58 ± 6.57	27.86 ± 8.71
% Pre-pregnancy hypertension	12.84% *p = 0.01*	0%
MAP at the first visit (mmHg)	89.44 ± 9.37	85.90 ± 7.19
Gestational age at delivery^a^ (wk)	37.76 ± 2.16	38.01 ± 1.09
% GDM	12.62%	36.36%
% Past history of HDP	16.22% *p* = 0.001	0%
% Non-medicated depression or anxiety	1.80%	0%

^a^mean ± SD

*BMI* body mass index, *MAP* mean arterial pressure, *GDM* gestational diabetes mellitus

## Discussion

We found that women who used antidepressant and/or anxiolytic drugs during the first and early second trimester had a 3-fold increased risk of PE compared to those who were not exposed, including a more than 6-fold increase for those using SNRI (aOR 6.46 [2.49–16.78]). Also, our results suggest that women who stopped their medication before the 16th week of gestation had a lesser at risk for PE as compared to those who pursued their medication after the 16th week. Adjustment for potential confounders did not change the observations.

Others studies suggested a link between antidepressant use and PE risk. Avalos et al. observed a significant increase in the risk of PE in women with a diagnosis of depression who took antidepressant medication during the second trimester (13–20 wks; *n* = 1732) compared to women without depression (*n* = 16,402) (adjusted risk relative (aRR): 1.70, _95%_CI: 1.30, 2.23) [26]. Toh et al. found that peri-conceptional SSRI use was associated with a higher risk of PE and that the PE risk was greater among women who continued treatment after the first trimester (15.2%, *n* = 14), compared with both non-users (2.4%, *n* = 135) and those who discontinued SSRIs (3.7%, *n* = 4) [29]. Using population-based health-care databases from British Columbia (69,448 pregnancies), Palmstem et al. suggested that women who used antidepressants were at increased risk of PE [28]. Our results based on a large single cohort confirm the association between antidepressant medication and preeclampsia risk. Moreover, the importance of the sample size (6000 participants) and the adjustment for many potential confounders reinforce the strength of the association.

Although we have adjusted for many confounding variables, we cannot exclude the possibility that other unidentified confounding variables may account for part of the observed association between antidepressant and/or anxiolytic medication and PE risk. One such variable that could not be taken into account was pregnancy-induced changes in the absorption and metabolism of antidepressants [37]. Substantial pharmacokinetic changes may occur during pregnancy for a number of commonly used antidepressants and mood stabilizers that may alter medication concentrations and thus contribute to drug response and/or adverse events [37]. Also, genetic variation in drug metabolizing enzymes, like cytochrome 2D6 and P450 (CYP2D6 and CYP450), may affect the metabolism of antidepressants and anxiolytics [11, 38].

Various mechanisms of action of antidepressants may be responsible for the increased risk of PE. Antidepressants act by altering the levels of neurotransmitters, such as serotonin, noradrenalin, norepinephrine and dopamine [39]. SSRI, SNRI and tricyclics inhibit serotonin transporters or both serotonin and norepinephrine transporters and consequently, augment extracellular concentrations of these monoamines [40]. Increased levels of vasoactive amines, including serotonin and catecholamines, have been found in women with PE [41]. These vasoactive amines play multiple roles, including in brain function, blood pressure control and immune responses [41]. In vitro and in vivo studies showed that serotonin and norepinephrine increased placental chorionic vein and umbilical artery vasoconstriction [42–45]. Antidepressant-mediated vasoconstriction could lead to uteroplacental underperfusion and ischemia [46]. Maternal vascular underperfusion is associated with PE [47] and it is believed that placental ischaemia-reperfusion injury is central to the development of PE [48]. Other studies suggested that SSRI drugs may inhibit the synthesis of nitric oxide, a vasodilator [49, 50]. In normal pregnancy, the decrease in systemic vascular resistance is thought to involve the presence of nitric oxide [48]. Therefore, antidepressants could prevent optimal adaptation of the vascular system in pregnancy, leading to an increase in peripheral vascular resistance and blood pressure elevation. A meta-analysis suggested that SNRI drugs, probably via their noradrenergic effects, can cause elevations of diastolic blood pressure [51].

### Limitations

Our study has some limitations. First, the prevalence of antidepressant and anxiolytic users was low (any trimester 4.9%; < 16 wks 3.2%). However, this rate was similar to findings from a Quebec Pregnancy Cohort in which Bérard et al. observed a 4.5% (4.3% during 1st trimester) proportion of antidepressant use during pregnancy [12]. Second, despite the size of the cohort, the number of women suffering from GH or PE and the proportion of antidepressant and/or anxiolytic users were also low, limiting the power of subgroups analyses. The effect of depression or anxiety and drug treatment of these mood disorders could not be separated completely. Of note, although not significant, women in the small subgroup with non-medicated depression or anxiety had an adjusted OR of 2.92, which suggests that maternal underlying disease may be, at least in part, the driver of the association seen between medication exposure in pregnancy and preeclampsia. Moreover, we didn’t have information concerning drug dosages, nor the severity of the mood disorders that were being treated. The information about the medication used during pregnancy was retrieved from a standardized prenatal follow-up form filled on each visit and included in medical records and was possibly in part self-reported by women during their follow-up visit. Also, the information concerning the proportion of women who were already taking antidepressant and/or anxiolytic medications before pregnancy was not available. It possible that some women taking antidepressants or anxiolytics during the first trimester were wrongly considered to have stopped if the medication information about others trimesters was poorly documented in the medical file. It is also possible that some women with mood or anxiety disorders were misclassified since some women may not have reported their mental distress to their health care professional. However, if there were women with mood or anxiety disorders in the non-users of antidepressant and/or anxiolytic drugs, the association found between antidepressant and/or anxiolytic users and preeclampsia risk would have been decreased, assuming that depression itself increases the PE risk. If it were the case, our results would be conservative. Also, unmeasured confounders, such unplanned pregnancy and pregnancy outcome history, could have had an impact on the observed association.

On the other hand, the homogeneity of our population is a major strength of our prospective study. With predominantly Caucasian (> 93%) women and a public health system where all pregnant women have access to similar pregnancy monitoring, the possibility of sampling bias is somewhat reduced. Our recruitment rate was 86%, which makes our cohort very representative of the pregnant women population of the Quebec City area. Conversely, homogenous samples hinder the external validity of the results and generalizability to other populations must be carefully examined. Finally, the findings from the current cohort study replicated previous findings regarding the main protective (smoking) and risk (BMI, hypertension, nulliparity, diabetes) factors for PE giving credibility to our main findings.

## Conclusions

In conclusion, compared to women unexposed to antidepressant/anxiolytic medication, depression and anxiety, those who were exposed to antidepressant and/or anxiolytic before the 16th week of their pregnancy were at increased risk of hypertensive disorders of pregnancy, in particular of PE, especially if they continued their medication until the third trimester. Even adjusted for several covariates, the risk of PE remained 3-fold higher in pregnant women using antidepressant and/or anxiolytic drugs. These women should receive special attention early in their pregnancy for their global risk of HDP. Additional studies will be needed to clarify if stopping medication may be beneficial for women using antidepressants and/or anxiolytics in early pregnancy.

## Abbreviations

BMI: Body mass index
CI: Confidence interval
GDM: Gestational diabetes mellitus
GH: Gestational hypertension
HBP: High blood pressure
HDP: Hypertensive disorders of pregnancy
MAP: Mean arterial pressure
MIP: Medical interruption of pregnancy
OR: Odd ratio
PE: Preeclampsia
RR: Risk relative
SD: Standard deviation
SNRI: Serotonin norepinephrine reuptake inhibitors
SSRI: Selective serotonin reuptake inhibitors
TCA: Tricyclic antidepressants
VIP: Voluntary interruption of pregnancy
Wk: Week
